# Effects of low-dose X-ray irradiation on activated macrophages and their possible signal pathways

**DOI:** 10.1371/journal.pone.0185854

**Published:** 2017-10-27

**Authors:** Jian Li, Zhen-yu Yao, Chang She, Jian Li, Bin Ten, Chang Liu, Shu-bin Lin, Qi-Rong Dong, Pei-Gen Ren

**Affiliations:** 1 Department of Orthopedics, The Second Affiliated Hospital of Soochow University, Suzhou, Jiangsu, China; 2 Department of Translational Medicine R&D Center, Shenzhen Institute of Advanced Technology, CAS, Shenzhen, Guangdong, China; University of Kansas Medical Center, UNITED STATES

## Abstract

Low-dose irradiation (LDI) has been used in clinics to treat human diseases, including chronic inflammation. This study assessed the effects of LDI on the inflammatory response of activated mouse primary peritoneal macrophages, and the underlying signal pathways. Primary peritoneal macrophages were isolated from mice and then incubated with lipopolysaccharide (LPS)-coated Ti microparticles (Ti-positive control) with or without brief exposure to LDI (X-ray, 0.5 Gy) 1 h later (Ti-LDI group) or left untreated in culture medium (Ti-negative control). The macrophages were then subjected to qRT-PCR, Western blot, cell viability CCK-8 assay, and ELISA. qRT-PCR analysis revealed the Ti-LDI group expressed significantly lower levels of IL-1β, IL-6, and TNF-α mRNA than those of the Ti-positive control group, while the ELISA data showed that Ti-LDI group had significantly lower secretion of IL-1β, IL-6, and TNF-α proteins. The most significant reduction associated with LDI was the secretion TNF-α protein, which barely increased from 13 to 25 h after treatment. Western blot data demonstrated that phosphorylation of p65 and ERK was much lower in the Ti-LDI group than in the controls. The data from the current study suggests that LDI of activated mouse macrophages was associated with significantly lower inflammation responses, compared with non-exposed activated macrophages, which was possibly through inhibition of the NF-κB and ERK pathways.

## Introduction

Inflammation and inflammation-related responses play an important role in the osteolysis around periprosthetic bone [1, 2] and inhibition of inflammation could help to effectively control osteolysis progression [3, 4]. To date, conventional treatments of inflammation are usually systematic and risk in developing a series of complications in a weakened immune system; thus, a more desirable therapeutic strategy to inhibit inflammation around the prosthesis would be local. For example, the local application of ionizing radiation, such as X-rays and gamma rays, is extensively used clinically. The effect of ionizing radiation on the human body is dependent on cell type, dose, exposure time, and a variety of other factors [5], e.g., in 1982, Luckey [6] first proposed that a low dose of ionizing radiation, or low-dose irradiation (LDI), was beneficial to animal health and survival. Since then, several previous studies have supported this hypothesis [7–9]. In particular, LDI was able to enhance osteoblast functions to promote mineralization [10], but significantly reduced oxidative burst in macrophages and reduced tissue inflammation [11]. Another *in vivo* study also determined that LDI was able to effectively reduce inflammation responses in arthritis and relieve symptoms [12].

Thus, development of stereotactic radiotherapy, especially with accurate positioning and faster dose attenuation, has allowed physicians to apply LDI for local treatment. To date, more than 40,000 LDI treatments were annually performed in Germany, mainly on patients with inflammatory disease or excessive pain and dysfunction due to inflammation [13]. We previously demonstrated [14] that LDI could promote osteoblast differentiation and increase periosteal osseointegration. LDI significantly reduced the thickness of the prosthetic pseudomembrane around the prosthesis *in vivo* [15]. Wear particles have also been reported to be among the most common causes for osteolysis around the prosthesis [14, 15]. They not only inhibit the proliferation and differentiation of osteoblasts and accelerate its apoptosis [16, 17], but also stimulate the inflammation cascade of macrophages and activate osteoclasts [17]. All these eventually lead to osteolysis around the prosthesis. However, to our best knowledge, there have been no reports regarding whether LDI can inhibit the inflammation response induced by wear particles. We thus, hypothesized in this study that proper regulation of bone metabolism by enhancing osteoblast function and inhibiting macrophage and osteoclast could be an effective way to prevent and treat periprosthetic osteolysis. In this study, we investigated the effects of LDI on the inflammatory response of macrophages activated by wear particles and explored the underlying possible signal pathways at the cell biology level. We first isolated primary peritoneal macrophages from mice and then assessed their purity using flow cytometry. We then determined the ratios of cell to Ti microparticle using the cell viability CCK-8 assay. After that, we selected 1:10 of the cell to Ti microparticle and incubated macrophages with lipopolysaccharide (LPS)-coated Ti microparticles (Ti-positive control) with or without brief exposure to LDI (X-ray, 0.5 Gy) 1 h later (Ti-LDI group) or left untreated in culture medium (Ti-negative control). Half of these activated cells were briefly receiving additional treatment of LDI at a dose of 0.5 Gy after the first hour incubation before resuming the incubation. The macrophages were then subjected to qRT-PCR, Western blot, and ELISA.

## Methods

### Preparation of titanium (Ti) suspension

Titanium (Ti) microparticles (D50 < 5 μm and D90 < 11.2 μm) were soaked in 10% Triton X-100 overnight after being washed with 75% ethanol for three times. The Ti microparticles were then baked at 180°C for 6 h after being rinsed with sterilized water. The baked Ti microparticles were further soaked in 75% ethanol for 48 h and then used to prepare a Ti suspension in phosphate-buffered solution (PBS) after be rinsed twice under sterilized conditions. The Ti suspension was incubated with 1 mg/mL of lipopolysaccharides (LPS) for 12 h at 37°C on a shaker.

LPS-coated Ti microparticles were obtained after the free LPS was removed by washing the resulting Ti microparticles for 10 times with PBS. The final LPS-coated Ti microparticle working suspension was prepared at 1.5 mg/mL using Dulbecco's modified Eagle's medium supplemented with 10% fetal bovine serum. This LPS-coated Ti microparticle working suspension was sealed and stored at 4°C until use.

### Isolation of mouse peritoneal macrophages

The animal protocol in this study was approved by the Institute Animal Care and Use Committee. Male Balb/c mice (6 week old, 25 ± 3 g) were purchased from the Animal Experimental Center of Soochow University (Soochow, China). The mice were housed at controlled room temperature (22 ± 1°C) and fed with standard mouse chow and were freely accessible to drinking water. To isolate peritoneal macrophages, five mice were first injected intraperitoneally with 2 mL per mouse of thioglycollate broth culture medium (Sigma Chemicals, St. Louis, MO, USA) according to a previous study [18] and 72 h later, the mice were euthanized and 10 mL of PBS was injected into the abdominal cavity of each mouse to wash out macrophages and was aspirated to collect peritoneal fluids. The cells were then counted after resuspended in the culture medium and we were usually able to obtain 8–12 × 10^6^ cells per mouse and approximately 90% were macrophages after flow cytometry. The cell suspensions were then seeded in 12-well plates at a density of 5 × 10^5^ macrophages per well or 6-well plates at a density of 1 × 10^6^ macrophages per well and cultured in an incubator with 5% CO_2_ at 37°C for 24 h and then subjected to further experiments.

### Flow cytometry

We performed the flow cytometry to assess the purity of isolated mouse peritoneal macrophages according to a previous study [18], i.e., isolated peritoneal macrophages were washed twice with PBS and then added with or without 1 μl each of monoclonal anti-F4/80 antibody (eBioscience, San Diego, CA, USA), anti-PE antibody (Thermo- Fisher, Waltham, MA, USA), and anti-CD11b antibody (Biolegend, San Diego, CA, USA) and incubated at 4oC for 30 min. The cells were then subjected to flow cytometric analysis (BD FACSria II, BD Biosciences, San Jose, CA, USA).

### Cell viability CCK-8 assay

The isolated peritoneal macrophages were washed twice with PBS and then seeded into 96-well plates at the density of 2 x 104 per well and incubated in an incubator with 5% CO_2_ at 37°C for 24 h. After that, the cells were treated with or without LPS-coated Ti microparticles at a ratio of 0, 1, 10, 50, 100, or 500 for 24 h. Next, the cells were washed with PBS three times to remove LPS-coated Ti microparticles and added with 10 μl of the CCK-8 solution (Beyotime, Shanghai, China) and further cultured for 2 h and the cell plates were centrifuged at 4000 rpm for 20 min and the supernatants were collected into a new plate, which was subjected to spectrophotometer analysis at 450 nM. The cells were in triplicate and the experiments were repeated at least once. The cell survival rate (%) was calculated using the formula of [A(Ti^+^)-A(control)]/ [A(Ti^-^)-A(#control)] x 100; A(Ti^+^), treated macrophages; A(control), no cells and no treatment with LPS-coated Ti microparticles; A(Ti^-^), macrophages without treatment with LPS-coated Ti microparticles.

### Macrophage treatments

Cultured cells were randomly divided into three groups, i.e., Ti-negative control, Ti-positive control, and Ti-LDI. In the Ti-negative control group, macrophages were incubated in the culture medium only without Ti microparticles and irradiation. The Ti-positive control and Ti-LDI groups were both incubated with a working concentration suspension of LPS-coated Ti microparticles with a cell-to-Ti microparticle ratio of 1:10, whereas the Ti-LDI group also received a brief X-ray LDI treatment at a dose of 0.5 Gy (200c Gy/min, 6 MV radiation source of medical linear accelerator X-ray irradiation for 15 seconds), 1 h post-incubation.

### qRT-PCR

At each time point of 3, 5, 9, 13, or 25 h post-incubation, macrophages from three wells of each group (Ti-negative control, Ti-positive control, and Ti-LDI) were subjected to isolation of total RNA using a Trizol reagent (maker, city, state) and then reversely transcribed into cDNA using the Primescript RT reagent kit (TaKaRa, Dalian, China) according to the manufacturers’ instructions. The resulted cDNA samples were subjected to qPCR amplification using the SYBR Premix Ex Taq II (TaKaRa) according to the manufacturer’s protocol.

Glyceraldehyde-3-phosphate dehydrogenase (GAPDH) was used as an internal control in the quantification of gene expression of interleukin (IL)-1β, IL-6, and tumor necrosis factor (TNF)-α for all 3 groups. For each gene, the gene expression data were normalized to that of the Ti-negative control group [16]. The primers used were: IL-1β, 5′-TGTGAAATGCCACCTTTTGA-3′ and 5′-TGTCCTCATCCTGGAAGGTC-3′; IL-6, 5′-CCGGAGAGGAGACTTCACAG-3′ and 5′-TCCAGTTTGGTAGCATCCATC-3′; TNF-α, 5′-CCACCACGCTCTTCTGTCTA-3′ and 5′-CACTTGGTGGTTTGCTACGA-3′; and GAPDH, 5′-CCAATGTGTCCGTCGT -3′ and 5′- GCGGAGATGATGACCCTTT -3′.

### Enzyme-linked immunosorbent assay (ELISA)

At 13 or 25 h post-incubation, cells from 3 wells of each group (Ti-negative control, Ti-positive control, and Ti-LDI) were randomly selected and the supernatants were collected for assessment of IL-1β, IL-6, and TNF-α levels using their ELISA assay kits (Biocompare, South San Francisco, USA) according to the manufacturer’s protocols. The microplates were analyzed at 450 nm using an ELISA plate reader (Tecan Group, Männedorf, Switzerland) to quantify the concentrations of each protein.

### Western blot

At 5, 9, or 13 h post-incubation, cells from 3 wells of each group were randomly selected to lyse using the immunoprecipitation cell lysis buffer for Western blot (Beyotime, Shanghai, China) containing a protease inhibitor (Abcam, Cambridge, UK). The protein concentrations were determined using a bicinchoninic acid assay kit (Beyotime) in accordance with the manufacturer’s instructions. The samples containing 20 μg proteins were resolved in 10% sodium dodecyl sulfate -polyacrylamide gel using electrophoresis and then transferred onto polyvinylidene fluoride membranes (Millipore, Billerica, MA, USA). For Western blotting, the membranes were blocked with 5% bovine serum albumin for 1 h at the room temperature and then incubated with antibodies against phospho-NF-κB pP65; NF-κB p65 (dilution 1:1000, Pathway Sampler Kit 9936, CST, USA); and phospho-ERK pP44/42; total ERK p44/42 (dilution 1:1000, Phospho-MAPK Family Antibody Sampler Kit 9910, CST, USA) overnight The anti-GAPDH antibody was used as the loading control. After incubation with each primary antibody, the membranes were further incubated with a secondary antibody (Cell Signaling Technology, Danvers, MA, USA) for 2 h at room temperature and scanned and quantified using a Gel Image Station (BIO-RAD, Hercules, CA, USA).

### Statistical analyses

Each experiment was performed in triplicate and repeated at least once or twice. Numerical data were expressed as mean ± standard error of the mean (SEM). All statistical analyses were performed using Graph Pad Prism 5.01 (San Diego, CA, USA) with the 2-tailed *t*-test. *P* < 0.05 was considered statistically significant.

## Results

### Isolation and assessment of purity of mouse peritoneal macrophages

**Fig 1:**
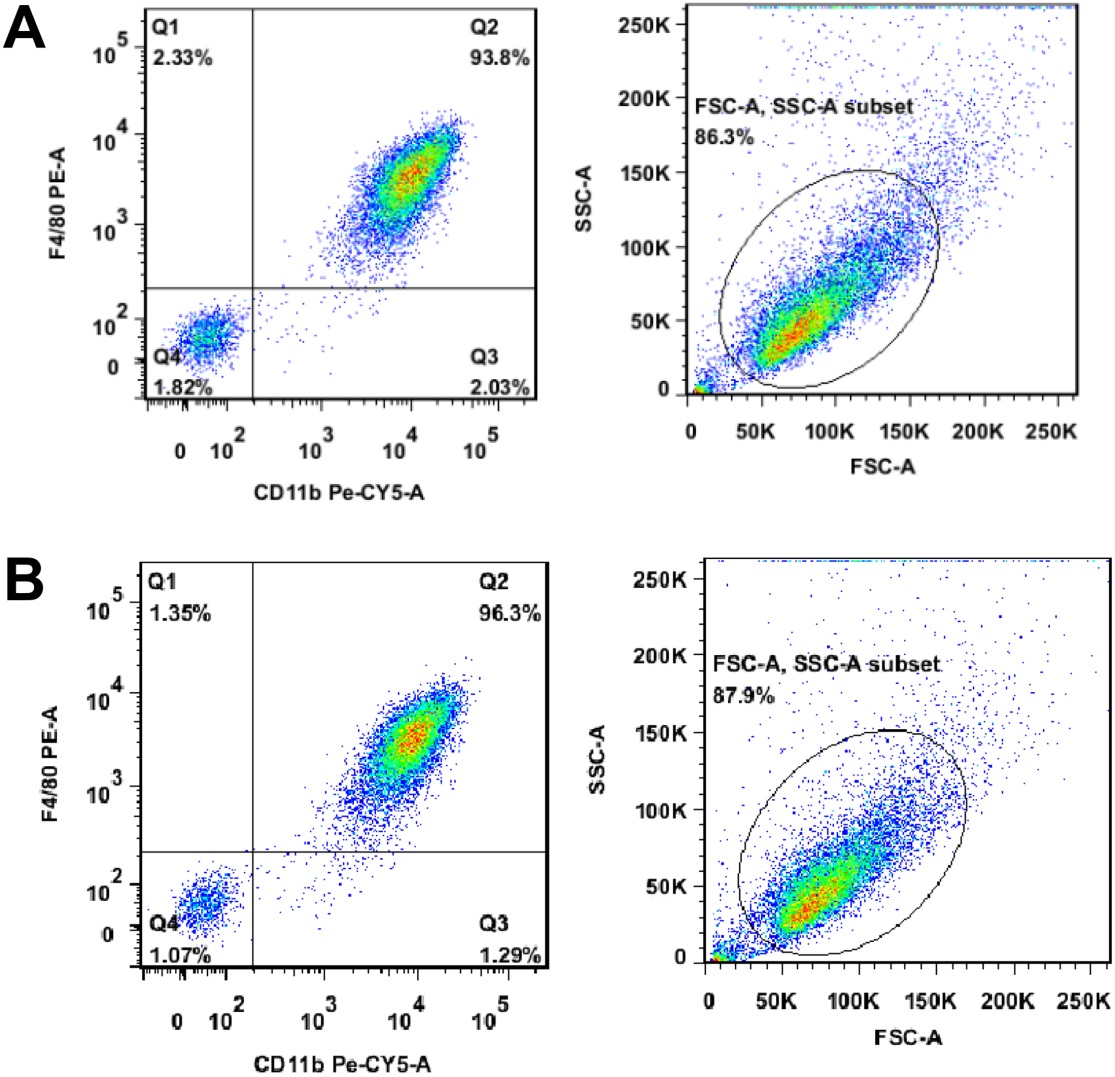
Assessment of mouse peritoneal macrophage purity. Mice were injected intraperitoneally with thioglycollate broth culture medium for 72 h and microphages were collected from the abdominal cavity and subjected to flow cytometric analysis of the purity using three different macrophage markers. (A) Macrophages directly from the abdominal cavity. (B) Macrophages used in our experiments.

We first injected five mice intraperitoneally with 2 mL per mouse of thioglycollate broth culture medium and 72 h later, the mice were euthanized and injected with 10 ml PBS into the abdominal cavity of each mouse to collect peritoneal fluids. After centrifugation and suspension in the culture medium, we counted and obtain approximately 8–12 × 10^6^ cells per mouse. We then performed flow cytometry and found the purity of the macrophages reached to 93.8% (Fig 1A and for our experiments, the macrophage purity was 96.3% (Fig 1B).

### Assessment of the working ratio of LPS-coated Ti microparticle to treat macrophages

**Fig 2:**
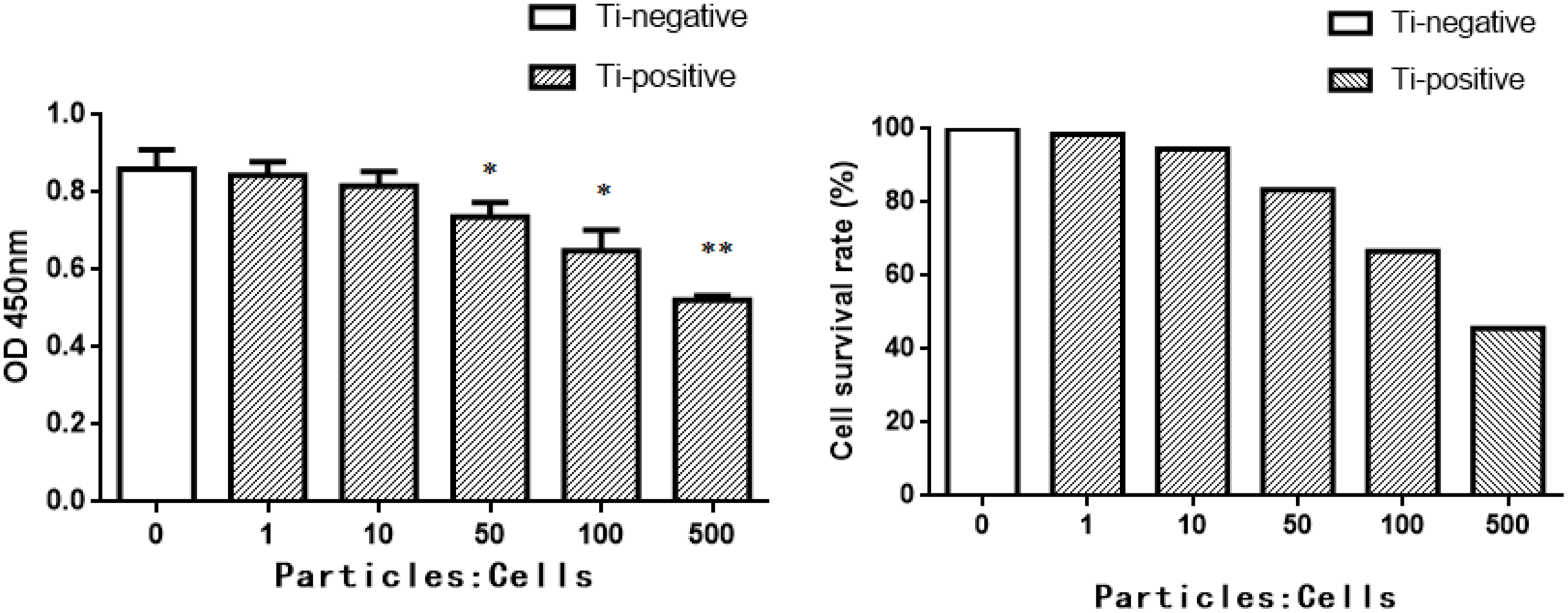
Viability of mouse peritoneal macrophages. Microphages were treated with or without LPS-coated Ti microparticles at a ratio of 0, 1, 10, 50, 100, or 500 for 24 h and then subjected to cell viability CCK-8 assay. Data are summarized and expressed as mean ± SEM. *P < 0.05 and **P < 0.01 comparing the Ti-negative with Ti-positive groups.

We then performed the cell viability CCK-8 assay on these macrophage samples to assess the best ratio of LPS-coated Ti microparticle on the macrophages and found that the ratios of 1:10 or below significantly affected cell viability, whereas the ratios of 1:10 or beyond showed that there was no significant change in cell viability (Fig 2). Thus, we selected the ratio of 1:10 for the following experiments.

### Effects of LDI on macrophage activation *in vitro*

**Fig 3:**
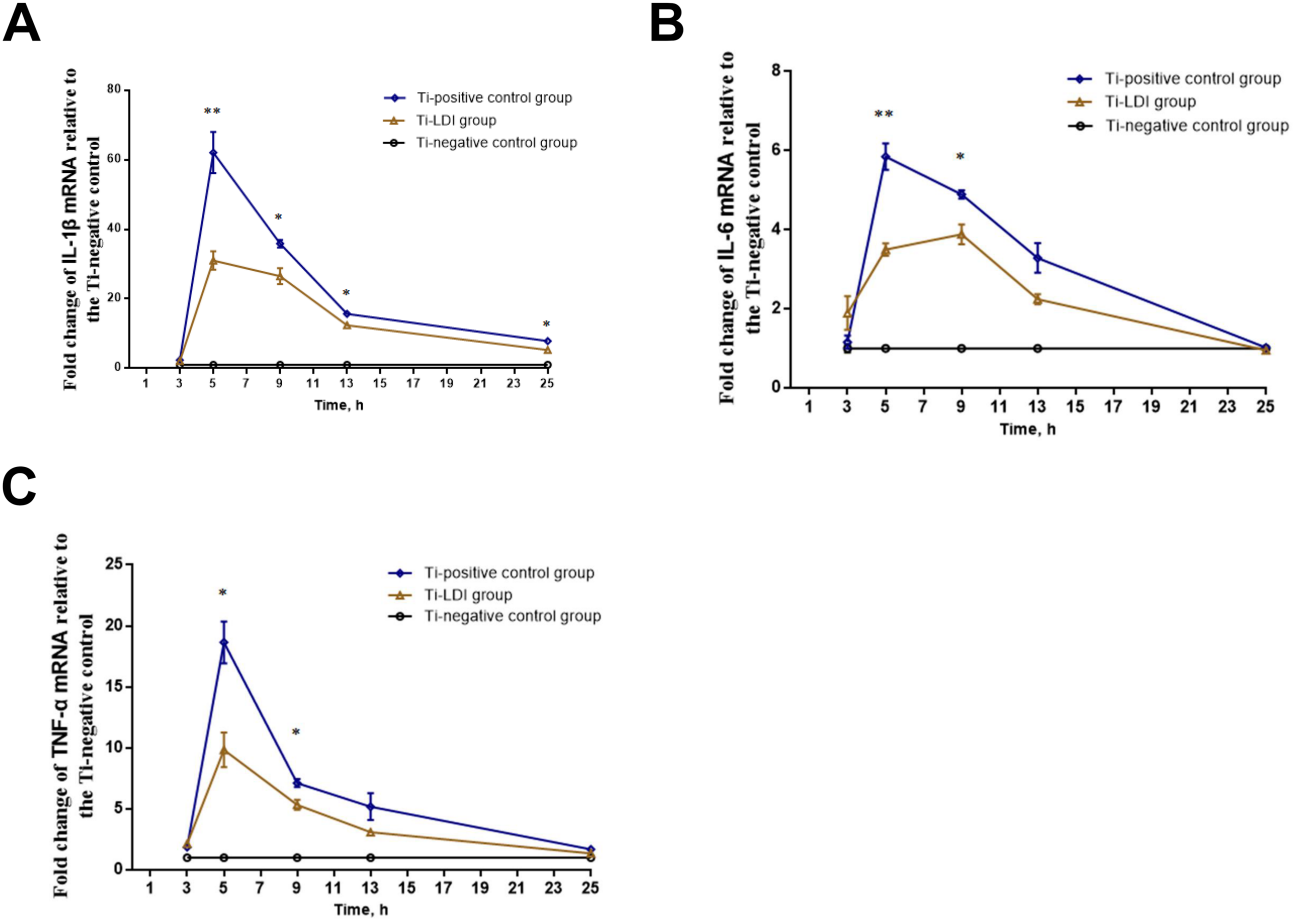
LDI suppression of the pro-inflammatory cytokine mRNA. (A) IL-1β. (B) IL-6. (C) TNF-α. Experiments were performed in triplicate and data are expressed as mean ± SEM. *P < 0.05 and **P < 0.01, comparing the Ti-positive control with Ti-LDI groups.

Compared with the cells in the Ti-negative control group, the cells that were incubated with LPS-coated Ti microparticles (i.e. both Ti-negative control and Ti-LDI groups) had significantly higher levels of all three pro-inflammatory transcripts of IL-1β, IL-6, and TNF-α at any time point starting from 5 h of incubation (Fig 3). However, the cells in the Ti-LDI group that received additional treatment of 0.5 Gy LDI had significantly lower levels of IL-1β, IL-6, and TNF-α mRNA at any time point starting from 5 h of incubation than those in the Ti-positive control group, i.e., level of IL-1β at 3 h_(0 Gy)_ was 2.35 ± 0.06, while level of IL-1β at 3 h_(0.5 Gy),_ was 2.06 ± 0.13 (p = 0.108). Level of IL-1β at 5 h_(0 Gy)_ was 62.07 ± 5.90 vs. at 5 h_(0.5 Gy)_ of 30.99 ± 2.65 (p = 0.0086); at 9 h_(0 Gy)_ of 35.87 ± 1.10 vs. at 9 h_(0.5 Gy)_ of 26.51 ± 2.30 (p = 0.021); at 13 h_(0 Gy)_ was 15.65 ± 0.81 vs. at 13 h_(0.5 Gy)_ of 12.36 ± 0.72 (p = 0.038); at 25 h_(0 Gy)_ was 7.82 ± 0.31 vs. at 25 h_(0.5 Gy)_ of 5.25 ± 0.60 (p = 0.019).

The level of IL-6 at 3 h_(0 Gy)_ was 1.16 ± 0.16, while level of IL-6 at 3h_(0.5 Gy)_ was 1.89 ± 0.42 (p = 0.18), whereas level of IL-6 at 5 h_(0 Gy)_ was 5.85 ± 0.33 vs. at 5 h_(0.5 Gy)_ of 3.50 ± 0.15 (p = 0.0031); at 9 h_(0 Gy)_ was 4.90 ± 0.11 vs. at 9 h_(0.5 Gy)_ of 3.88 ± 0.25 (p = 0.02); at 13 h_(0 Gy)_ was 3.29 ± 0.37 vs. at 13 h_(0.5 Gy)_ of 2.24 ± 0.13 (p = 0.057); ay 25 h_(0 Gy)_ of 1.02 ± 0.06 vs. at 25 h_(0.5 Gy)_ of 0.95 ± 0.07 (p = 0.52). Moreover, the level of TNF-α at 3 h_(0 Gy)_ was1.87 ± 0.02, while the level of TNF-α at 3 h_(0.5 Gy)_ was 2.14 ± 0.11 (p = 0.07), whereas level of TNF-α at 5 h_(0 Gy)_ was 18.66 ± 1.71 vs. at 5 h_(0.5 Gy)_ of 9.83 ± 1.42 (p = 0.016); at 9 h_(0 Gy)_ was 7.12 ± 0.34 vs. at 9 h_(0.5 Gy)_ of 5.33 ± 0.41 (p = 0.028); at 13 h_(0 Gy)_ was 5.19 ± 1.09 vs. at 13 h_(0.5 Gy)_ of 3.10 ± 0.13 (p = 0.13); at 25 h_(0 Gy)_ was 1.69 ± 0.13 vs. at 25 h_(0.5 Gy)_ of 1.35 ± 0.06 (p = 0.068).

**Fig 4:**
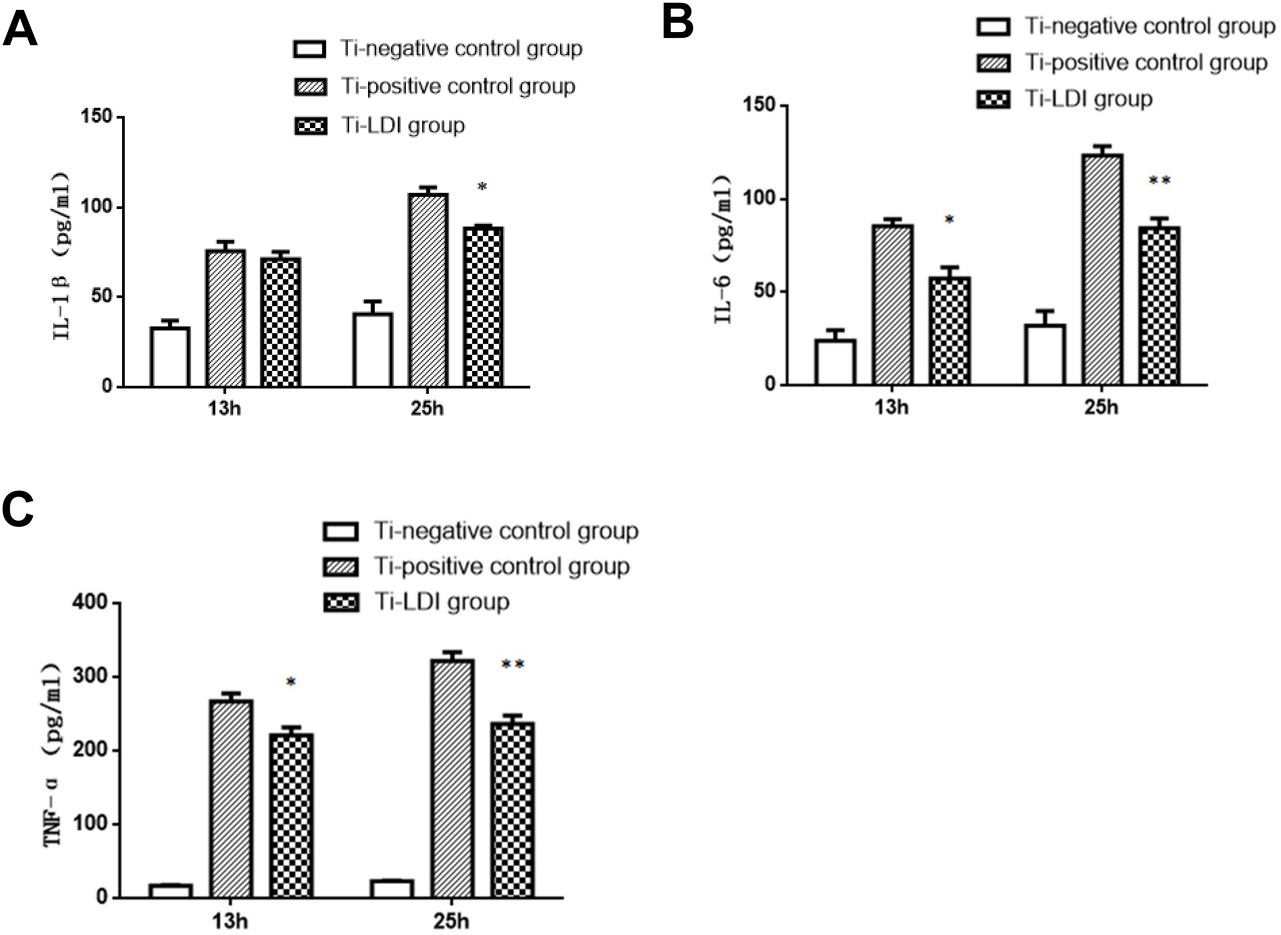
LDI suppression of pro-inflammatory cytokine secretion. (A) IL-1β. (B) IL-6. (C) TNF-α. Experiments were performed in triplicate and data are expressed as mean ± SEM. *P < 0.05 and **P < 0.01, comparing the Ti-positive control with Ti-LDI groups.

We further analyzed expression of their proteins using ELISA and found that the secretion of IL-1β, IL-6, and TNF-α proteins was enhanced after the stimulation of macrophages with LPS-coated Ti microparticles (Fig 4). However, treatment with 0.5 Gy irradiation significantly attenuated the increase in the secretion of IL-1β, IL-6, and TNF-α proteins, i.e., IL-1β at 13 h_(0 Gy)_ was 75.63 ± 5.15 pg/ml vs. IL-1β level at 13 h_(0.5 Gy)_ of 71.01 ± 4.33 pg/ml (p = 0.53), whereas IL-1β level at 25 h_(0 Gy)_ was 107.0 ± 4.04 pg/ml vs. at 25 h_(0.5 Gy)_ of 88.16 ± 1.60 pg/ml (p = 0.012). Moreover, level of IL-6 protein at 13 h_(0 Gy)_ was 85.26 ± 3.83 pg/ml vs. at 13 h_(0.5 Gy)_ of 57.31 ± 5.94 pg/ml (p = 0.017); at 25 h_(0 Gy)_ was 123.3 ± 5.02 pg/ml vs. at 25 h_(0.5 Gy)_ of 84.35 ± 5.22 pg/ml (p = 0.0058). In addition, the level of TNF-α protein at 13 h_(0 Gy)_ was 266.8 ± 10.59 pg/ml vs. at 13 h_(0.5 Gy)_ of 220.4 ± 11.20 pg/ml (p = 0.04); at 25 h_(0 Gy)_ was 321.5 ± 11.93 pg/ml vs. at 25 h_(0.5 Gy)_ of 236.0 ± 11.85 pg/ml(p = 0.0071).

### Effects of LDI on regulation of gene expression in macrophage *in vitro*

**Fig 5:**
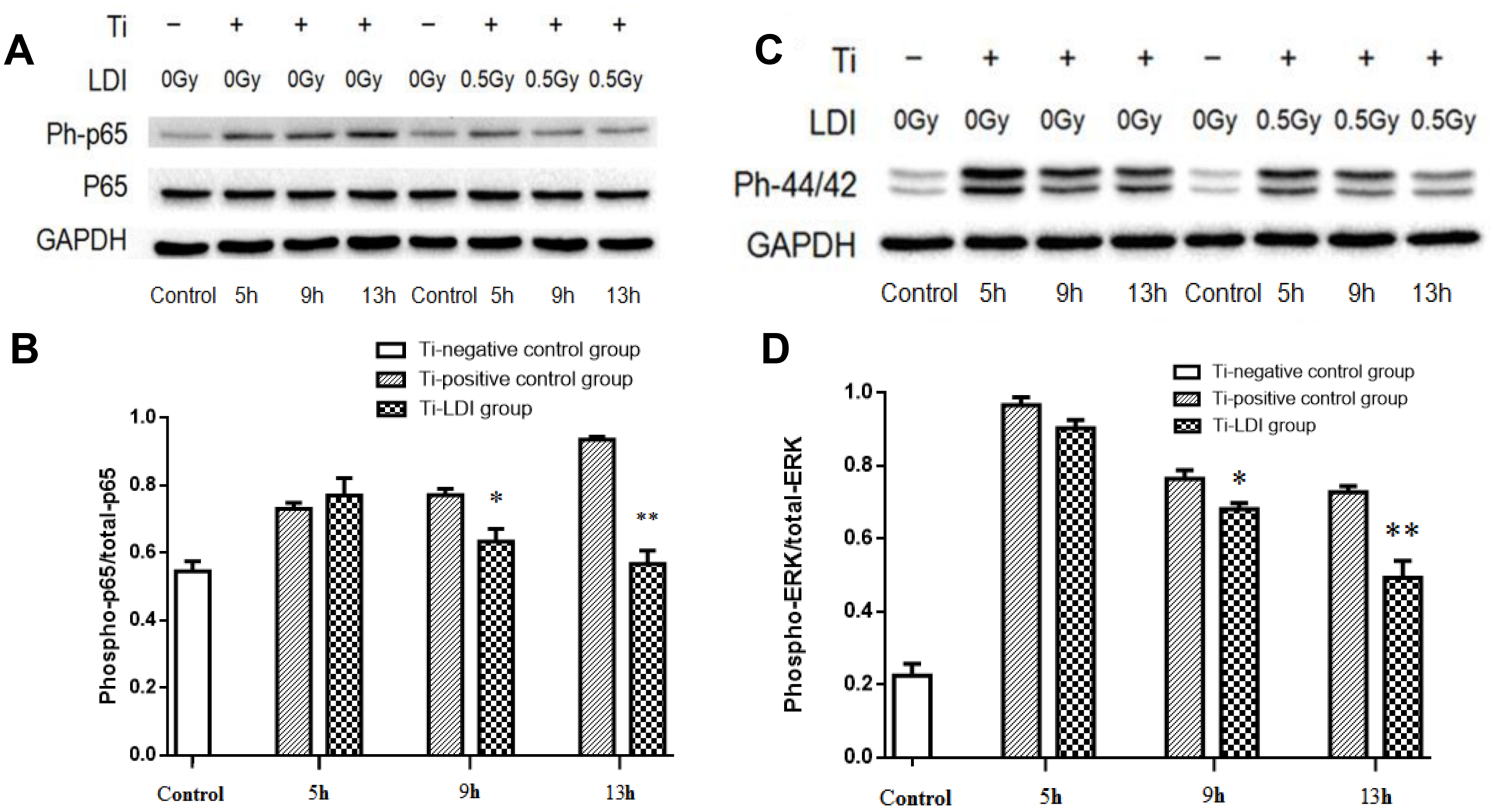
LDI suppression of NF-κB and ERK1/2 phosphorylation. (A) NF-κB Western blot. (B) The ratio of p-p65 and p65. (C) ERK1/2 Western blot. (D) The ratio between p-ERK1/2 and total ERK1/2. Experiments were performed in triplicate and data are expressed as mean ± SEM. *P < 0.05 and **P < 0.01, comparing the Ti-positive control with Ti-LDI groups.

We then performed Western blot experiments to confirm incubation with LPS-loaded Ti microparticles to increase levels of p65 phosphorylation but treatment with irradiation significantly inhibited p65 phosphorylation (Fig 5AB). This effect became more obvious up on duration of treatment, i.e., the ratio of p-p65/p65 at 5 h_(0 Gy)_ 0.7312 ± 0.018 vs. at 5 h_(0.5 Gy)_ of 0.7703 ± 0.052 (p = 0.51); at 9 h_(0 Gy)_ was 0.7718 ± 0.019 vs. at 9 h_(0.5 Gy)_ of 0.6343 ± 0.038 (p = 0.031); at 13 h_(0 Gy)_ was 0.9365 ± 0.009 vs. at 13 h_(0.5 Gy)_ of 0.5674 ± 0.040 (p = 0.0009). Furthermore, the simulation also upregulated phosphorylation of ERK protein (Fig 5CD). However, this effect was significantly inhibited by 0.5 Gy irradiation, i.e., ratio of the pERK/tERK at 5 h_(0 Gy)_ was 0.9668 ± 0.022 vs. at 5 h_(0.5 Gy)_ of 0.9032 ± 0.023 (p = 0.119); at 9 h_(0 Gy)_ was 0.7662 ± 0.025 vs. at 9 h_(0.5 Gy)_ of 0.6813 ± 0.017 (p = 0.047); and at 13 h_(0 Gy)_ was 0.7284 ± 0.017 vs. at 13 h_(0.5 Gy)_ of 0.4931 ± 0.047 (p = 0.0092).

## Discussion

Previous studies have shown that the inflammation responses induced by wear particles were strongly associated with the endotoxin attached to the microparticles [19, 20]. Our pre-experimental data also revealed that LPS-uncoated Ti microparticles had very limited effects of macrophages (S1 Fig). Clinically, it has been reported that patients could have aseptic loosening of the prosthesis due to bacterial components, such as endotoxin [21, 22] in the absence of infection symptoms. Therefore, in the present study, we stimulated the macrophages with LPS-coated Ti microparticles, the average particle size of approximately 5 μm. These microparticles are similar in size to wear particles occurring under physiological conditions and can be endocytosed by macrophages [23] and sufficiently affect their functions [24].

In the current study, we isolated macrophages from mouse peritoneal cavity after injected thioglycollate-containing medium. A previous study [18] revealed that such an isolation could contaminate up to 20% non-macrophage, among which eosinophils could significantly inhibit activation of p38 MAPK and Akt [18]. However, our current data showed that the purity of macrophages was more than 90% and additionally, our current study compared data on Ti-LPS-coated microparticles with or without LDI; thus, a little level of non-macrophages may not affect our experimental data.

Indeed, it has been reported that local X-ray irradiation at a dose of 0.5 Gy is an effective treatment for acute necrotizing inflammation [25]. In the present study, we thus, irradiated activated mouse primary peritoneal macrophages with X-rays at a dose of 0.5 Gy. This low-dose X-ray irradiation did not have immediate effects on the inflammatory factors IL-1β, IL-6, and TNF-α levels in macrophages, as showed at 2 h post-irradiation (Fig 3). However, the cells in the Ti-LDI groups had statistically significant lower level of IL-1β, IL-6, and TNF-α mRNA starting at 5 h of incubation than in the cells that were only incubated with LPS-coated Ti microparticles without receiving the additional LDI treatment (Fig 3). We also observed that the increase in secretion of these inflammatory factors was significantly slower in the Ti-LDI group during the incubation period of 13–25 h (Fig 4). In particular, an increase in only approximately 16 pg/mL was observed for the most sensitive factor, TNF-α in macrophages after treatment.

Although LDI possesses beneficial biological effects on tissues, it has also been reported that LDI may cause micro-damage to cells in the early stage by increase in formation of reactive oxygen species (ROS) [26], DNA double-strands breaks [27], or chromosomal breakage [28]. These may have contributed to the results of the present study regarding gene expression and secretion of inflammatory factors during the course of the first 2 h after irradiation. However, another explanation may be that the biological effects of LDI occur more slowly than do the effects associated with LPS-coated Ti microparticles.

Inhibition of the inflammatory response can alleviate osteolysis induced by wear particles. However, clinically the inhibition of a single inflammatory factor could not produce any obvious improvement in osteolysis [29], due to a possible compensatory increase in other inflammatory factors. Therefore, effective treatment strategies should target the upstream signaling pathway of the inflammatory responses [4]. For example, NF-κB is one of the most important transcription factors in cells and possesses important biological functions, such as the immune and inflammatory responses, apoptosis, and tumorigenesis. In present study, LPS-coated Ti microparticles enhanced phosphorylation of p65 protein in macrophages and the proportion of phosphorylated p65 protein increased with time in macrophages after stimulated by LPS-coated Ti microparticles (i.e., the Ti-positive control group). This may be attributed to the positively-looped inflammatory cascade triggered by LPS-coated Ti microparticles. However, it is worthy to note that LDI did not immediately (i.e. within 4 h post-irradiation) inhibit the activation of NF-κB induced by LPS-coated Ti microparticles. The inhibitory effect of LDI on NF-κB activation was observed only at 8 h after irradiation. Thereafter, this inhibitory effect became more obvious as time passed, which might be due to the blocked inflammatory cascade. It is possible that a therapeutic effect of LDI on granulosa-induced periprosthetic osteolysis may be achieved by blocking the NF-κB signaling pathway.

Furthermore, mitogen-activated protein kinase (MAPK) is also important in physiological and pathological processes of many cells, such as regulation of cell growth and differentiation, adaption to the environmental stress, and inflammation reactions. Among the MAPK pathways, the ERK pathway is the important one in proliferation and differentiation of osteoclasts. Our experimental data showed that LDI was able to reduce ERK1/2 phosphorylation in macrophages. This observation is consistent with the reports by other researchers regarding the ERK pathway in inflammatory osteolysis [30, 31]. Therefore, the anti-inflammatory effect of LDI may be related to manipulation of the ERK pathway.

## Conclusions

In conclusion, 0.5 Gy LDI significantly inhibited inflammation responses in mouse primary peritoneal macrophages by reduction and inhibition of IL-1β, IL-6, and TNF-α expression and secretion, effect of which could be related to the simultaneous inhibition of both the NF-κB and ERK pathways.

## Supporting information

S1 Fig No effects of LPS-uncoated Ti microparticle on induction of the pro-inflammatory cytokine mRNA level. (A) IL-1β. (B) IL-6. (C) TNF-α. The experiments were performed in triplicate and data are expressed as mean ± SEM. *P < 0.05 and **P < 0.01, comparing the Ti-LPS-coated group with Ti-negative control group.
(TIF)
