# Supplementary figures and images for: Effects of low-dose X-ray irradiation on activated macrophages and their possible signal pathways
Source: PLoS One. 2017 Oct 27;12(10):e0185854. doi: 10.1371/journal.pone.0185854 (PMC5659615; doi:10.1371/journal.pone.0185854)

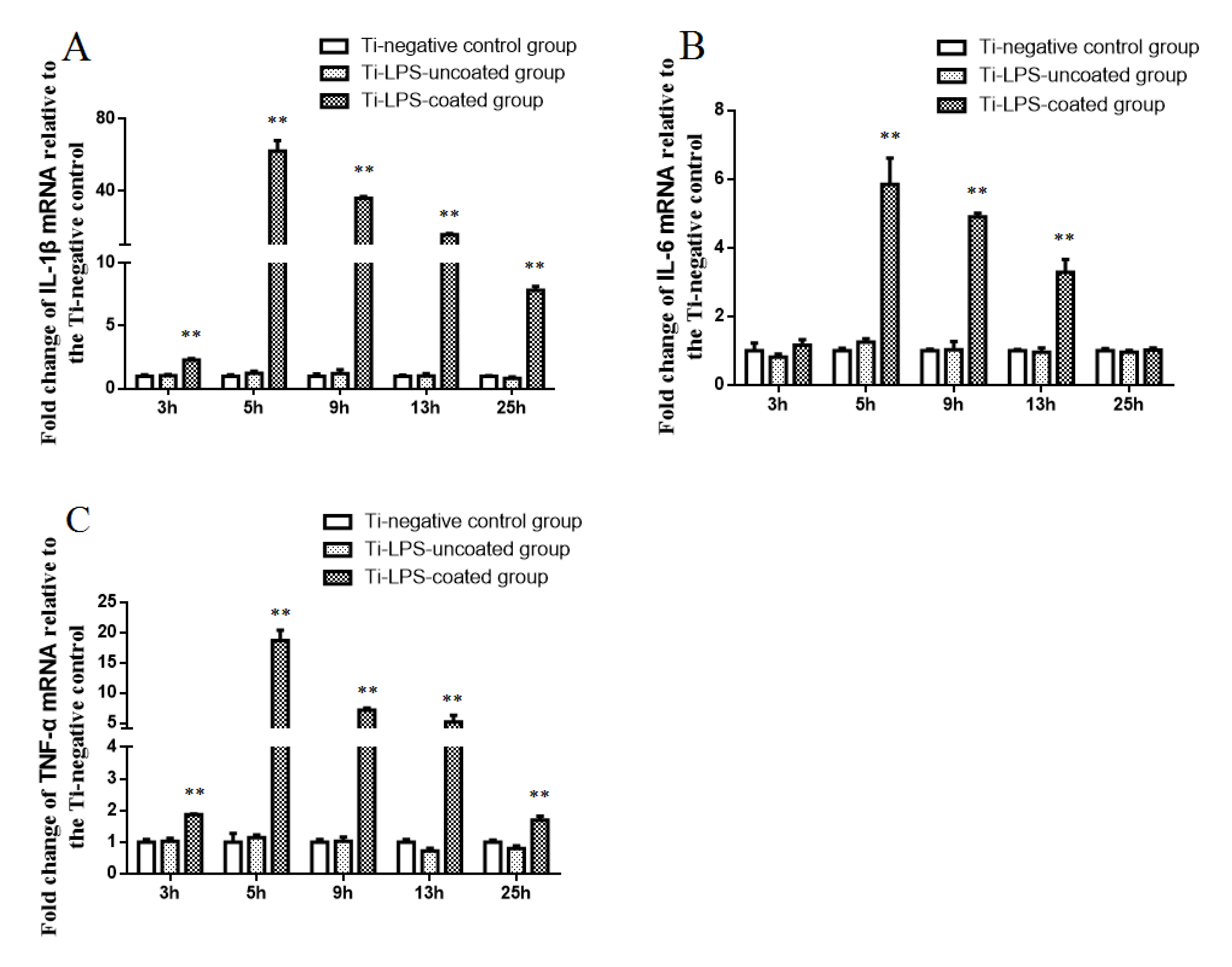

Supplement: S1 Fig — (A) IL-1β. (B) IL-6. (C) TNF-α. The experiments were performed in triplicate and data are expressed as mean ± SEM. *P < 0.05 and **P < 0.01, comparing the Ti-LPS-coated group with Ti-negative control group. (TIF) [file pone.0185854.s001.tif]
